# VSP-17, a New PPARγ Agonist, Suppresses the Metastasis of Triple-Negative Breast Cancer via Upregulating the Expression of E-Cadherin

**DOI:** 10.3390/molecules23010121

**Published:** 2018-01-08

**Authors:** Yuhui Wang, Menglin Zhu, Bo Yuan, Kefeng Zhang, Mingli Zhong, Wei Yi, Xiaotian Xu, Xiaoqun Duan

**Affiliations:** 1 Guangxi Colleges and Universities Key Laboratory of Pharmacology, Guilin Medical University, 109 Huanchengbei Road Two, Guilin 541004, China; wangyuhuitg2017@163.com (Y.W.); zmlvarar123456@126.com (M.Z.); yuanbo3917@163.com (B.Y.); zkftg2017@163.com (K.Z.); zmltg2017@163.com (M.Z.); 2 Shanghai Institute of Materia Medica, Chinese Academy of Sciences, Shanghai 201203, China; 18607739261@163.com

**Keywords:** VSP-17, triple-negative breast cancer, metastasis, E-cadherin, PPARγ, agonist

## Abstract

Triple-negative breast cancer (TNBC), an aggressive subtype of breast cancer, shows higher metastases and relapse rates than other subtypes. The metastasis of TNBC is the main reason for the death of TNBC patients. Increasing evidence has shown that inhibiting the metastasis of TNBC is a good method for TNBC treatment. Here, VSP-17 was designed and synthesized as an agonist of PPARγ, evidenced by upregulating the expression of CD36 and increasing the activity of PPARγ reporter gene. VSP-17 obviously inhibited the migration and invasion process of MDA-MB-231 cells but showed little effect on the viability of MDA-MB-231 cells. Notably, VSP-17 could selectively promote the expression of E-cadherin without affecting the expression of BRMS1, CXCL12, MMP9, Orai1, Stim1, TGF-β, and VEGF. In addition, VSP-17 significantly suppressed the metastasis of liver and promoted the expression of E-cadherin in MDA-MB-231 xenograft model. In conclusion, VSP-17 inhibited the metastasis process of TNBC via upregulating the expression of E-cadherin.

## 1. Introduction

Triple-negative breast cancer (TNBC), an aggressive subtype of breast cancer, is characterized by deficiency of estrogen receptor (ER), progesterone receptor (PR), and human epidermal growth factor receptor-2 (HER2). The global breast cancer mortality is declining, which is partially due to progress in early diagnosis of breast cancer, but the TNBC subtype is still the main cause of human death according to “Cancer Statistics, 2017” [1]. Until now, the metastasis and relapse of TNBC are the major clinical features in the late stage of TNBC and constitute the direct causes of death [2,3]. Current studies suggest that the inhibition of metastasis might be an effective approach for the successful treatment of TNBC [4]. Therefore, agents that have an anti-metastasis effect are valuable for TNBC treatment.

TNBC metastasis, a stage of breast cancer cells where the disease has spread to distant sites beyond the blood vessel and the axillary lymph nodes. The main steps involved in the metastatic cascade of breast cancer cells are listed as follows: firstly, cells divide from the primary tumor and then invade the primary tumor barrier surrounding the breast cancer cells. After the intravasation into the circulatory system through the bloodstream or lymph channels, cells then extravasate to a distant site and proliferate at the metastatic site of breast cancer cells. Increasing evidence has shown that the process of migration and invasion plays key roles in breast cancer metastasis and that these processes can be regulated by a variety of factors, such as BRMS1, E-cadherin, and VEGF [5,6,7,8,9]. Therefore, the abrogation of aberrant factors expression might inhibit the migration and invasion process of TNBC and lead to the inhibition of TNBC metastasis.

Thiazolidinediones, the class of drugs known as peroxisome proliferator-activated receptor-γ (PPARγ) agonists, were considered effective drugs for breast cancer metastasis [10,11,12,13]. Increasing evidence has shown that rosiglitazone, one of the thiazolidinediones, can inhibit the migration and invasion of breast cancer cells by regulating many signaling pathways [14,15,16,17]. However, the long-term usage of rosiglitazone may cause adverse reactions, such as weight gain, fluid retention, and an increase in the risk of heart disease [18,19,20]. Therefore, developing a new type of PPARγ agonist with high selectivity might be a better strategy for the metastasis of TNBC. In light of the advantages of the structure of rosiglitazone and SR1664 [21], VSP-17 was designed and synthetized by our research group. The present study aimed to explore the underlying mechanism for the anti-metastasis effect of VSP-17, with special emphasis on the expression of E-cadherin.

## 2. Results

### 2.1. The Synthetic Routes of VSP-17

**Figure 1:**
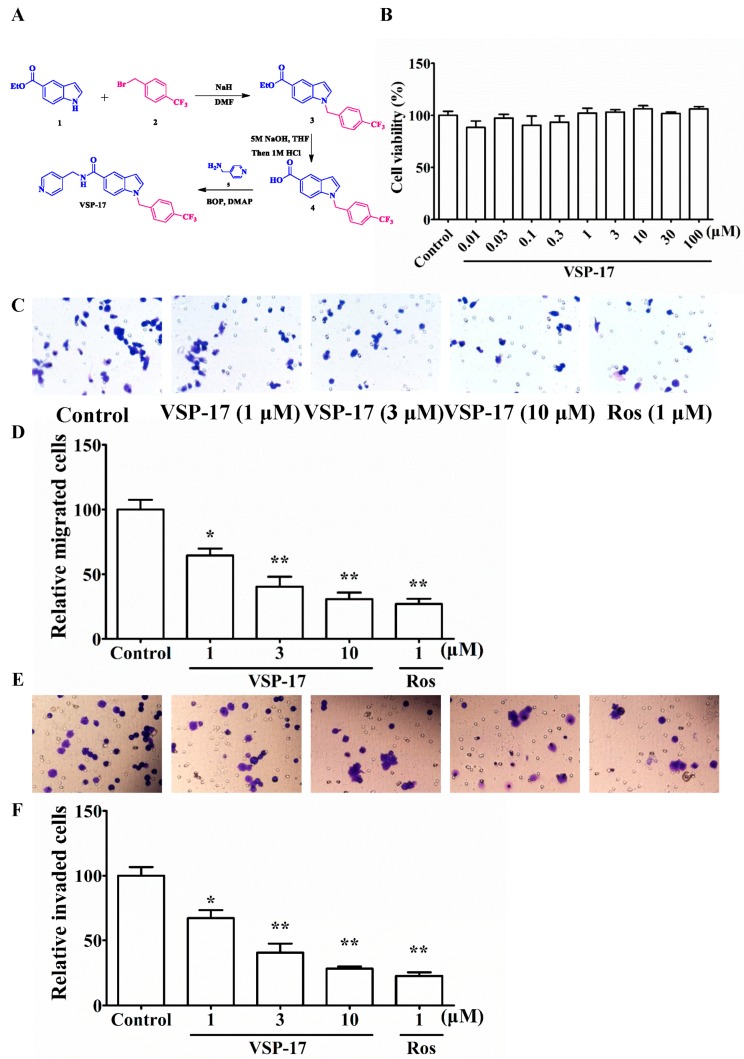
Synthesis of VSP-17 and effect of VSP-17 on MDA-MB-231 cell migration and cell invasion. (**A**) Synthesis of VSP-17; (**B**) Effect of VSP-17 on MDA-MB-231 cell viability; (**C**,**D**) Effect of VSP-17 on MDA-MB-231 cell migration. Cell migration was detected by using cell migration assay; (**E**,**F**) Effect of VSP-17 on MDA-MB-231 cell invasion. MDA-MB-231 cells were cultured in chambers with matrigel followed by treatment with VSP-17 (1, 3, 10 μM) for 24 h. The number of cells invaded through the matrigel of chambers bottom were counted in three different regions. The data were expressed as the means ± S.E.M. of three independent experiments. * *p* < 0.05, ** *p* < 0.01 vs. control.

As shown in Figure 1A, (i) NaH (60% dispersion in mineral oil, 11.0 mmol) was added in portions at 0 °C to a stirred solution of methyl 1*H*-indole-5-carboxylate **1** (1.89 g, 10.0 mmol) in dry DMF (10 mL). After stirring for 30 min at 0 °C, 1-(bromomethyl)-4-(trifluoromethyl) benzene **2** (2.88 g, 12.0 mmol) was added and the mixture was then stirred at room temperature for 24 h. Then, the reaction mixture was cooled to ambient temperature, poured into H_2_O (100 mL), and extracted with EtOAc (100 mL). The organic phase was dried over anhydrous Na_2_SO_4_. After evaporation of the solvents under reduced pressure, the crude product was purified on a silica gel column using EtOAc/petroleum ether (1:9) to obtain the pure **3** (2.94 g, 80%) as a white solid. ^1^H NMR (400 MHz, CDCl_3_) *δ* 8.29 (d, *J* = 1.6 Hz, 1*H*), 7.76–7.65 (m, 4*H*), 7.55 (d, *J* = 8.7 Hz, 1*H*), 7.36 (d, *J* = 7.9 Hz, 2*H*), 6.74–6.66 (m, 1*H*), 5.60 (s, 2*H*), 3.83 (s, 3*H*); (ii) A suspension of **3** (1.67 g, 5.0 mmol) and 5 M NaOH solution (5 mL) in THF (20 mL) was stirred at 50 °C until consumption of the starting material. It was allowed to reach room temperature, 2 M HCl was added, diluted with EtOAc (50 mL) and washed brine. The combined organic phase was dried (Na_2_SO_4_). After evaporation of the solvents under reduced pressure, the crude product was purified on Flash chromatography to afford the product **4** (1.41 g, 88%) as a white solid. ^1^H NMR (400 MHz, CDCl_3_) *δ* 12.47 (s, 1*H*), 8.26 (s, 1*H*), 7.76–7.66 (m, 3*H*), 7.65 (d, *J* = 3.2 Hz, 1*H*), 7.51 (d, *J* = 8.6 Hz, 1*H*), 7.36 (d, *J* = 8.1 Hz, 2*H*), 6.68 (d, *J* = 3.1 Hz, 1*H*), 5.60 (s, 2*H*), 1.39 (s, 2*H*); (iii) To a solution of **4** (1.60 g, 5.0 mmol) in dry DMF (20 mL) were added 2.0 equiv. of DMAP (1.22 g, 10 mmol) and 2.0 equiv. of BOP (4.42 g, 10 mmol) at 0 °C in an ice bath. After stirring for 0.5 h, 1.2 equiv. of pyridin-4-ylmethanamine **5** (0.65 g, 6.0 mmol) were added, and the reaction was allowed to stir until all starting material had disappeared. Then, the mixture was poured into a 10% aqueous solution of HCl. The aqueous phase was extracted with EtOAc (2 times), and the combined organic layers were dried (anhydrous Na_2_SO_4_) and concentrated in vacuo. Flash chromatography (Silica gel, hexane/ethyl acetate = 2/1) yielded the desired product VSP-17 (1.64 g, 80%) as a colorless solid. ^1^H NMR (400 MHz, CDCl_3_) *δ* 8.59–8.51 (m, 2*H*), 7.83–7.77 (m, 2*H*), 7.65 (d, *J* = 8.1 Hz, 2*H*), 7.58–7.52 (m, 2*H*), 7.25–7.22 (m, 2*H*), 7.04–6.96 (m, 2*H*), 6.63 (t, *J* = 6.0 Hz, 1*H*), 5.17 (s, 2*H*), 4.64 (d, *J* = 6.0 Hz, 2*H*). HRMS (ESI) *m/z* calcd. for [M + H]^+^: 410.1480, found: 410.1478 [M + H]^+^.

### 2.2. Effect of VSP-17 on the Cell Viability of MDA-MB-231 Cells

MTT assay was used to determine the effect of test compounds on the cell viability and proliferation in tumor cells. The results showed that VSP-17 had little cytotoxicity on MDA-MB-231 cells at concentrations below 10 μM (Figure 1B). Therefore, VSP-17 (1, 3, 10 μM) could be used for further experiments.

### 2.3. Effect of VSP-17 on the Migration and Invasion of MDA-MB-231 Cells

Migration and invasion are important processes of metastasis. To determine the anti-metastasis effect of VSP-17, the effects of VSP-17 on the migration and invasion of MDA-MB-231 cells were investigated by transwell methods. As shown in Figure 1C,D, VSP-17 suppressed the migration of MDA-MB-231 cells in a concentration-dependent manner. Moreover, the invasion of MDA-MB-231 cells was also inhibited by VSP-17 treatment in a concentration-dependent manner (Figure 1E,F). In conclusion, these findings suggested that the repression of migration and invasion played pivotal roles in the VSP-17-mediated inhibition of MDA-MB-231 cells metastasis.

### 2.4. The Key Role That E-Cadherin Plays in the Anti-Migration and Anti-Invasion Effect of VSP-17

**Figure 2:**
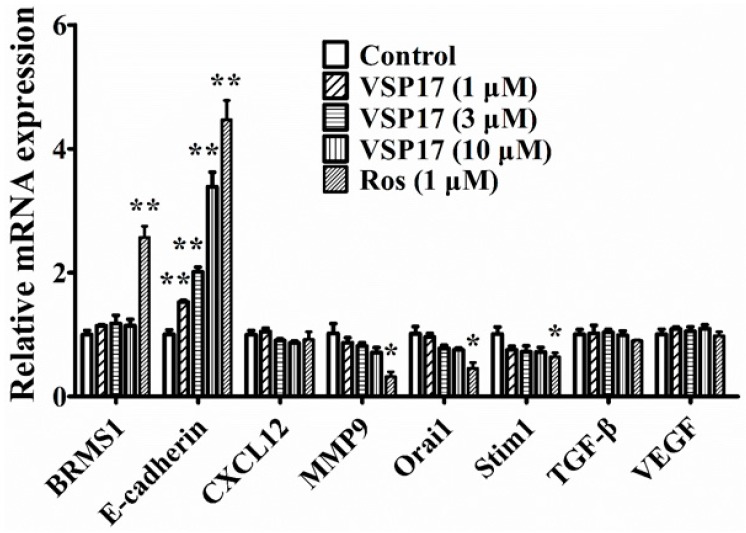
Effect of VSP-17 on metastasis-related factors in MDA-MB-231 cells. MDA-MB-231 cells were treated with VSP-17 (1, 3, and 10 μM) for 24 h, and the mRNA expressions of breast cancer metastasis suppressor 1 (BRMS1), E-cadherin, CXCL-12, metal matrix proteinase 9 (MMP9), calcium release-activated calcium channel protein 1 (Orai1), stromal interaction molecule 1 (Stim1), transforming growth factor (TGF-β), and vascular endothelial growth factor (VEGF) were measured using a Q-PCR assay. The data were expressed as the means ± S.E.M. of three independent experiments. * *p* < 0.05, ** *p* < 0.01 vs. control.

The migration and the invasion processes of tumor cells can be regulated by many factors, such as BRMS1, E-cadherin, CXCL12, MMP9, Orai1, Stim1, TGF-β, and VEGF [22,23,24,25,26,27,28]. To identify the mechanisms in anti-migration and anti-invasion effect of VSP-17, the expression of migration and invasion-associated factors above were studied. As shown in Figure 2, VSP-17 (1, 3, and 10 μM) upregulated the mRNA expression of E-cadherin in a concentration-dependent manner in MDA-MB-231 cells. In contrast, VSP-17 showed a slight effect on the mRNA expression of BRMS1, CXCL12, Orai1, Stim1, TGF-β, and VEGF in MDA-MB-231 cells. These results suggested that E-cadherin might play a vital role in the VSP-17-mediated inhibition of migration and invasion in MDA-MB-231 cells.

### 2.5. The Effect of VSP-17 on the Metastasis Markers and Liver Metastasis in MDA-MB-231 Xenograft Model

**Figure 3:**
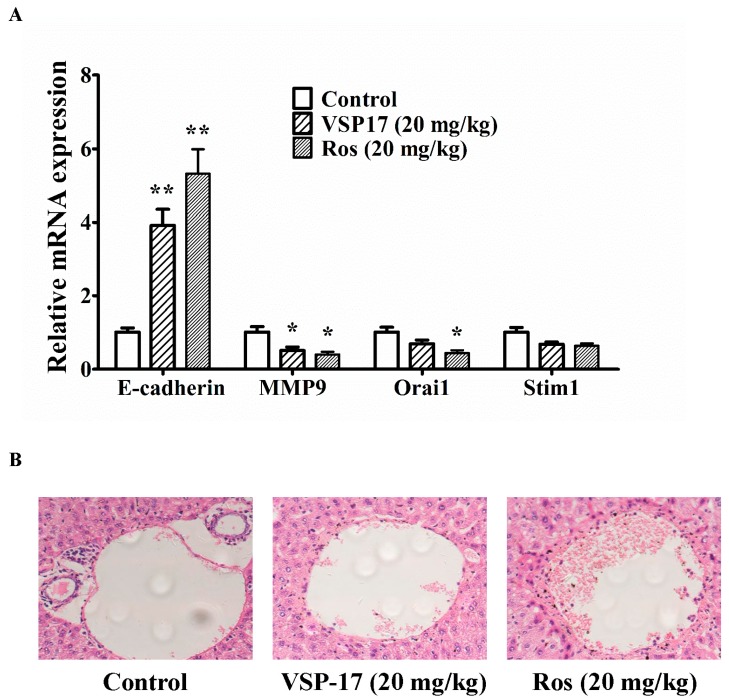
Effect of VSP-17 on liver metastasis of MDA-MB-231 xenograft model in vivo. Mice were injected with MDA-MB-231 cells at the left and right mammary fat pads, and then treated with VSP-17 (20 mg/kg) and rosiglitazone (20 mg/kg) for five weeks. (**A**) Tumor samples from breast of mice were collected at the end of five weeks and subjected to Q-PCR assays for E-cadherin, MMP9, Orai1, Stim1 mRNA expression; (**B**) H&E staining was used for detecting metastatic tumor cells in mouse livers. Arrows indicated infiltration of tumor cells. The data were expressed as the means ± S.E.M. of three independent experiments, *n* = 6. * *p* < 0.05, ** *p* < 0.01 vs. control.

To confirm the upregulation effect on E-cadherin and consequent anti-metastasis activity of VSP-17, a xenograft model was established by subcutaneous injection of MDA-MB-231 cells into the left and right mammary fat pads of nude mice. Tumor samples emerged in the breasts of mice in model group, VSP-17-treated groups (20 mg/kg) and rosiglitazone-treated groups (20 mg/kg) were used to determine the effect of VSP-17 on the expression of metastasis-related factors. It was shown that the expression of E-cadherin was lower in the tumors in the model group. Compared with model mice, VSP-17 (20 mg/kg) and rosiglitazone (20 mg/kg) treatments enhanced the expression of E-cadherin at transcriptional level in MDA-MB-231 xenografts mice (Figure 3A). Moreover, the mRNA expression of MMP9 was downregulated by VSP-17 (20 mg/kg) and rosiglitazone (20 mg/kg) treatments.

The metastasis from the injection sites to the liver was studied by histological examination at seven weeks after MDA-MB-231 cells injection. The infiltration of tumor cells was observed in the liver of mice in the model group. It is worth noting that no or less infiltration was detected in the liver of VSP-17- and rosiglitazone-treated mice. The inhibition potency of VSP-17 (20 mg/kg) on the metastasis of MDA-MB-231 was superior to that of rosiglitazone (20 mg/kg) (Figure 3B). Taken together, these findings indicted that VSP-17 suppressed the metastasis of TNBC by upregulation of E-cadherin and a consequent anti-metastasis effect.

### 2.6. VSP-17 Could Activate PPARγ

**Figure 4:**
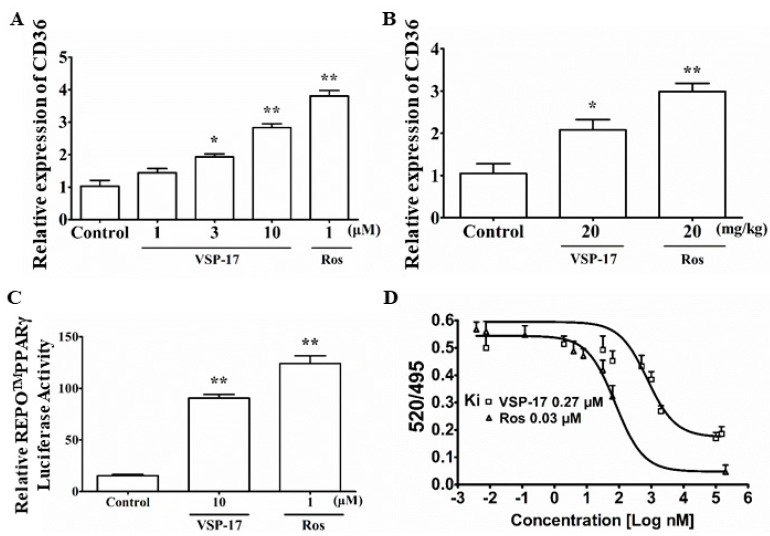
Effect of VSP-17 on the activation of PPARγ. (**A**) Effect of VSP-17 on the expression of CD36 in MDA-MB-231 cells. The cells were treated with VSP-17 (1, 3, 10 μM) and rosiglitazone (1 μM) for 24 h. The mRNA expression of CD36 was detected using Q-PCR analysis; (**B**) Effect of VSP-17 on the expression of CD36 in MDA-MB-231 xenograft model. Mice were injected with MDA-MB-231 cells at the left and right mammary fat pads and then treated with VSP-17 (20 mg/kg) and rosiglitazone (20 mg/kg) for five weeks. Tumor samples from the breasts of mice were collected at the end of five weeks and subjected to Q-PCR analysis for CD36 expression; (**C**) Effect of VSP-17 on PPARγ reporter gene activity. MDA-MB-231 cells were transiently transfected with REPO^TM^PPARγ and then subjected to indicated treatments for 24 h. Cells were then harvested and assayed for luciferase activity. Rosiglitazone is an agonist of PPARγ. GW9662 is the antagonist of PPARγ; (**D**) Binding of VSP-17 to PPARγ-LBD in a competitive TR-FRET assay. Data were expressed as means ± S.E.M. of three independent experiments. * *p* < 0.05 vs. control, ** *p* < 0.01 vs. control.

Subsequently, we observed whether VSP-17 could activate PPARγ. As shown in Figure 4A,B, the results showed that VSP-17 enhanced the expression of CD36 (a target gene of PPARγ) in MDA-MB-231 cells and MDA-MB-231 xenograft mice. Furthermore, VSP-17 elevated PPARγ reporter gene activity in MDA-MB-231 cells (Figure 4C). In addition, a time-resolved fluorescence resonance energy transfer (TR-FRET) assay further demonstrated that VSP-17 bounded to PPARγ with a kinetic inhibition constant (Ki) of 0.27 μM (Figure 4D). These data suggested that VSP-17 could activate PPARγ.

## 3. Discussion

The two primary hallmarks of TNBC are the excessive proliferation and high metastasis of breast cancer cells. Increasing evidence has shown that the metastasis of TNBC cells mainly involves invasion and migration, which are considered key steps in the metastasis process. VSP-17, a new synthetic compound, was proved as a highly selective PPARγ agonist with lower toxicity and high efficiency. In the present study, VSP-17 clearly inhibited the migration and invasion in MDA-MB-231 cells. The potential mechanisms were subsequently elaborated. These results substantially deepen our understanding of the anti-metastasis effect of VSP-17 on the metastasis of TNBC; this anti-metastasis effect effectively protected against TNBC and other subtypes of metastatic breast cancers.

Metastasis is a stage of breast cancer where the disease has spread to distant sites beyond the axillary lymph nodes. The process of migration and invasion are an important step in the metastasis of breast cancer, and these processes can be regulated by a variety of factors such as BRMS1, E-cadherin, CXCL12, MMP9, Orai1, Stim1, TGF-β, and VEGF. The changed expression of these factors is closely associated with the poor prognosis of TNBC patients [29,30]. Therefore, the abrogated expression of the aberrant factors may inhibit the migration and invasion of TNBC. Our study showed that VSP-17 inhibited the migration and invasion of MDA-MB-231 cells in vitro and prevented liver metastasis of the MDA-MB-231 xenograft model in vivo. In addition, VSP-17 upregulated the mRNA expression of E-cadherin but not the other metastasis-related factors, indicating that E-cadherin played key roles in VSP-17-mediated inhibition of MDA-MB-231 cells migration and invasion.

PPARγ, a ligand-dependent transcription factor that can regulate fatty acid storage and glucose metabolism, plays a vital role in the proliferation, apoptosis, and metastasis of TNBC cells. Increasing evidence has indicated that PPARγ is non-activated in TNBC cells and its activation might inhibit the metastasis of breast cancer cells. Therefore, PPARγ activation might be beneficial in TNBC therapy. Rosiglitazone, one of the thiazolidinediones, can inhibit metastasis in a murine model of hepatocellular carcinoma [31] and inhibit pancreatic cancer cell invasion and metastasis by regulating MMP-2 expression through phosphatase and tensin homolog (PTEN) [32]. There is also growing evidence that PPARγ agonist pioglitazone can inhibit NF-κB activation in cisplatin nephrotoxicity by reducing p65 acetylation via the AMPK-SIRT1/p300 pathway [33]. All these findings suggested that PPARγ agonists have great potential as anti-metastasis drugs for TNBC. However, the long-term use of rosiglitazone can cause a few adverse reactions, such as weight gain, fluid retention, and an increase in the risk of heart disease. Therefore, developing a new type of PPARγ agonist with high selectivity might be a better strategy for TNBC metastasis treatment. Combining rosiglitazone and SR1664, VSP-17 was designed and synthesized as a PPARγ agonist by our team. We showed that PPARγ was involved in the VSP-17-mediated upregulation of E-cadherin and the consequent inhibition of metastasis in MDA-MB-231 cells. The result showed that VSP-17 could promote the expression of CD36 (a target gene of PPARγ) in MDA-MB-231 cells and MDA-MB-231 xenograft tumors. The TR-FRET assay further demonstrated that VSP-17 had the ability to bind PPARγ with a binding constant of 0.27 μM. In addition, VSP-17 was shown to increase reporter gene activity in a PPARγ-dependent manner, implying that VSP-17 might be an agonist of PPARγ.

In conclusion, the inhibition of migration and invasion is a major effect of VSP-17, which in turn works to inhibit the metastasis of breast cancer, and it functions by upregulating the expression of E-cadherin.

## 4. Materials and Methods

### 4.1. Reagents

VSP-17 (C_23_H_18_F_3_N_3_O, MW: 409.1402, purity ≥ 98%) was designed and synthesized by our group (Figure 1A); LanthaScreen™ TR-FRET PPARγ Competitive Binding Assay was purchased from Thermo Fisher Co., Ltd. (Thermo Fisher, MA, USA); fetal bovine serum (FBS) was obtained from PAA (Linz, Germany). TRIzol reagent was obtained from Invitrogen (Carlsbad, CA, USA). Rosiglitazone (a PPARγ agonist) was obtained from Sigma Co., Ltd. (St. Louis, MO, USA). Other analytical reagent grade chemicals were obtained from Sinopharm Chemical Reagent Co., Ltd. (Nanjing, China).

### 4.2. Cell Culture

MDA-MB-231 cells were obtained from American Type Culture Collection (ATCC, Manassas, VA, USA) and cultured in a humidified incubator at 37 °C under 5% CO_2_ atmospheric condition in corresponding medium supplemented with 10% FBS, 100 U/mL streptomycin, and 100 U/mL penicillin.

### 4.3. Cell Migration Assay

Cell migration assay was carried out by using transwell plates (Millipore, Billerica, MA, USA) according to manufacturer protocol. MDA-MB-231 cells were treated with VSP-17 (1, 3, 10 μM) or rosiglitazone (1 μM) for 6 h, and detached and suspended in culture medium. Then, cells (1 × 10^4^ cells/well) was added into the upper chamber of the transwell plates, while the lower chamber was filled with 600 μL of culture medium with 10% FBS as a chemoattractant. After being incubated for 6 h at 37 °C, the non-migrated cells on the upper surface of the membrane were removed with a soaked cotton swab. In addition, the cells that migrated to the bottom face of the membranes were counted after being stained with crystal violet solutions. Then, fields per filter were captured randomly at a magnification of 200× with Olympus IX51 inverted microscope (Olympus, Tokyo, Japan).

### 4.4. Cell Invasion Assay

Cell invasion assay was performed by using a transwell chamber with a 10 mm diameter and an 8 μm pore size polycarbonate membrane (Corning Costar, Cambridge, UK) coated with matrigel. MDA-MB-231 cells were treated with VSP-17 (1, 3, 10 μM) or rosiglitazone (1 μM) for 24 h, and detached and suspended in culture medium. An aliquot (200 μL) of cells (1 × 10^5^ cells/mL) was added into the upper chamber of the transwell, while the lower chamber was filled with 600 μL of medium with 10% FBS as a chemoattractant. After being incubated for 24 h at 37 °C, the non-invaded cells on the upper surface of the membrane were removed with a soaked cotton swab, and the cells that invaded the bottom face of the membranes were counted after being stained with crystal violet solutions. Then, fields per filter were captured randomly at a magnification of 200× with Olympus IX51 inverted microscope.

### 4.5. Quantitative Real-Time Polymerase Chain Reaction (Q-PCR)

MDA-MB-231 cells were treated with VSP-17 or rosiglitazone, and the total RNA were isolated by using TRIzol reagent according to the supplier’s instructions. The cDNA was transcribed from RNA using a HiScript RT Super Mix (Vazyme, Nanjing, China) and then analyzed for expressions of E-cadherin, breast cancer metastasis suppressor 1 (BRMS1), metal matrix proteinase 9 (MMP9), calcium release-activated calcium channel protein 1 (Orai1), stromal interaction molecule 1 (Stim1), CXCL12, TGF-β, and VEGF with an Ace Q-PCR SYBR Green Master Mix (Vazyme, Nanjing, China) and with the help of MyiQ2 Detection System (Bio-Rad Laboratories, Hercules, CA, USA).

### 4.6. Luciferase Reporter Assay

MDA-MB-231 cells were incubated in 96-well plates (1 × 10^4^ cells/mL), and the cells in each well were co-transfected with PPRE-REPO. A PPRE-driven luciferase reporter plasmid was applied for examining specific activation of PPARγ binding to the PPRE. The cells were suspended in fresh culture medium and exposed to VSP-17 (1, 3, 10 μM) and rosiglitazone (1 μM) in a combination for 12 h. Then, the cells were lysed, and the supernatants were collected. The luciferase activity was measured by a luciferase assay system and a multimode reader.

### 4.7. PPARγ Competitive Binding Assay

The LanthaScreen time-resolved fluorescence resonance energy transfer (TR-FRET) PPARγ competitive binding assay was applied according to the manufacturer’s protocol. VSP-17 or rosiglitazone was cultured with GST-fused human PPARγ-LBD, terbium-labeled anti-GST antibody, and a fluorescently labeled PPAR ligand for 3 h in the dark at room temperature. The FRET signal was valued by excitation at 340 nm and emission at 520 nm for fluorescein and 495 nm for terbium. The ability of binding to the PPARγ-LBD was measured by the downregulation of the 520/495 nm ratio.

### 4.8. Animals and Treatment

Female athymic nude mice, 4–6 weeks old, were obtained from Cavens Laboratory Animals Co., Ltd. (Changzhou, China). All animal experiments were performed in strict accordance with the Animal Ethics Committee of Guilin Medical University and the animal permit number is GLMC201703011.

MDA-MB-231 cells re-suspended in PBS (1 × 10^7^ cells/mL) were injected subcutaneously into the left and right mammary fat pads of mice. Two weeks after initial implantation, mice were divided into three groups: (1) control group; (2) VSP-17 (20 mg/kg) group; (3) rosiglitazone (20 mg/kg) group. VSP-17 and rosiglitazone were orally administered three times per week for the duration of the experiment.

All animals were sacrificed after five weeks of treatments, and the tumors from the breasts of mice were collected and utilized for the detection of RNA using Q-PCR, respectively.

### 4.9. Statistical Analysis

The data were presented as the means ± S.E.M. Statistical analysis was performed using one-way analysis of variance (ANOVA) followed by Tukey’s test. *p*-values less than 0.05 (*p* < 0.05) were accepted as a significant difference.
